# The Effect of Domestic Sewage Treatment on Islands Using Ecological Treatment Processes: A Case Study of Haimen Island, Fujian Province

**DOI:** 10.3390/ijerph192315440

**Published:** 2022-11-22

**Authors:** Yuanmin Sun, Kunxian Tang, Hui Song, Degang Jiang, Shan Chen, Wulin Tu, Luchun Cai, Haiping Huang, Fei Zhang

**Affiliations:** 1 Third Institute of Oceanography, Ministry of Natural Resources, Xiamen 361005, China; 2 Key Laboratory of Marine Ecological Conservation and Restoration, Ministry of Natural Resources, Xiamen 361005, China; 3 Fujian Provincial Key Laboratory of Marine Ecological Conservation and Restoration, Xiamen 361005, China; 4 Institute of Marine Science and Technology, Shandong University, Qingdao 266237, China; 5 Island Research Center, Ministry of Natural Resources, Pingtan 350400, China

**Keywords:** island, sewage treatment, constructed wetland, ecological pond

## Abstract

Islands are characterized by a lack of land and freshwater resources, public finances, and technical personnel. As a result, domestic sewage may not be effectively treated, which can lead to major pollution on islands and in the surrounding sea areas. In this study, a pilot treatment of domestic sewage was conducted using an ecological treatment process (i.e., a constructed wetland and ecological pond) in an abandoned pond located on Haimen Island, Fujian Province, China. The pollutant indicators were monitored to analyze this treatment method at different treatment stages. The results showed that the combination of multiple ecological treatment processes had favorable treatment effects on various pollutants in the sewage. The treatment rates of the chemical oxygen demand (COD_Cr_) and suspended solids (SS) surpassed 88%. The treatment rate of the biochemical oxygen demand (BOD_5_), ammonia nitrogen (NH_3_-N), total nitrogen (TN), total phosphorus (TP), and fecal coliform surpassed 93%, and all the indicators met or were close to the level I B emission standards for urban sewage treatment plants. Different treatment stages have different treatment effects on different pollutants. The constructed wetland played an important role in sewage treatment through plant absorption, substrate adsorption, sedimentation, and microbial decomposition, particularly for the TP, COD_Cr_, and BOD_5_. In contrast, algal photosynthesis in the ecological pond produced a large amount of dissolved oxygen, and the treatment effect was highest for the TN and NH_3_-N. The treatment effects on the fecal coliform in the constructed wetland and ecological pond were very significant. Ecological treatment processes based on the combination of a constructed wetland and ecological pond have favorable treatment effects, low construction and maintenance costs, and pollution-free conditions, which are suitable for application in island areas.

## 1. Introduction

In recent decades, as the intensity of island development has increased, the amount of domestic sewage produced on islands has also increased. However, due to the low level of economic development on many islands and their lack of financial resources, their domestic sewage is often not effectively treated; instead, it is directly discharged, resulting in pollution on the islands and in the surrounding sea [1,2]. Domestic sewage on islands primarily comes from domestic water, which typically contains high levels of nitrogen, phosphorus, and organic matter [3]. Untreated sewage is discharged into ditches and ponds and can eventually reach the sea, leading to eutrophication in the area [4]. In China, sewage treatment facilities in urban areas are becoming increasingly effective, but on village-level islands, sewage treatment facilities are relatively lacking.

At present, sewage treatment technology includes physical methods [5], chemical methods [6], and ecological methods. Among them, physical methods cannot continuously and thoroughly treat polluted water, and chemical methods degrade aquatic ecosystems [7]. Ecological methods can economically and effectively remove pollutants from sewage through the natural anaerobic and aerobic processes of microorganisms, plants, and substrates [8,9,10]. Due to their advantages, such as low construction and operating costs, a minimal need for manual participation, and a good pollutant-removal efficiency [11], ecological methods have been increasingly used for various types of sewage treatment [12,13], particularly for island areas with less developed economies and a lack of technical personnel [1]. Common ecological methods include constructed wetlands [14,15], ecological ponds [16], and ecological floating beds [17], which can be used alone or in combination.

Due to a shortage of freshwater resources and low rates of domestic water consumption, the concentration of pollutants in domestic sewage is higher than that of most land areas. The objective of this study was to determine whether effective treatment could be achieved by using only one of the ecological treatment methods. In addition, we assessed the changing abundance of bacteria and viruses during sewage treatment. Microbes play an important role in the reduction in fecal coliform and inhibition of algal blooms in ecological ponds. Therefore, in this study, a village-level island in southern Fujian Province was selected as a case study. A pond that receives sewage and waste was transformed into a constructed wetland and ecological pond (including ecological floating beds), and pollutants were monitored at various stages of the treatment system. The mechanism of combined ecological treatment and its application prospects in island areas were investigated.

## 2. Materials and Methods

### 2.1. Experimental Location

**Figure 1:**
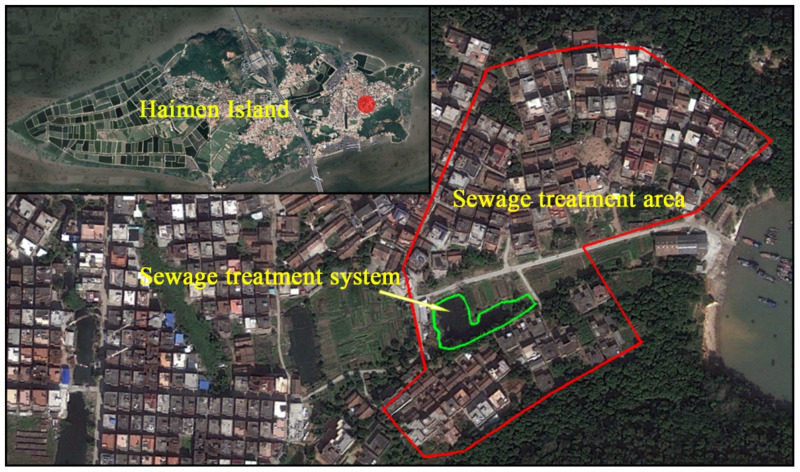
Location of the study area.

The study area was located on Haimen Island, which is situated in the eastern part of Zhangzhou City, Fujian Province (24°24′38.25″ N, 117°58′24.10″ E). The island has a population of 6300. The villagers make a living through fishing, mariculture, and farming. The domestic sewage on the island is directly discharged into a sewage ditch without treatment, collected into a pond or ditches by the village, and eventually discharged into the surrounding sea. The ditches and surface waters through which the sewage flows are severely polluted, the water becomes black and malodorous, and the ponds are extremely eutrophic. The odor generated by sewage pollutes the surrounding air and mosquitos and flies breed in the water, affecting the lives of residents. In addition, the anti-seepage capacity of the surface sewage ditch is poor, leading to serious groundwater pollution. Based on tests, the concentration of NH_3_-N in the well water consumed by the island residents is relatively high, and the concentration of dissolved oxygen (DO) is very low. Therefore, the need for domestic sewage treatment on Haimen Island is urgent. The domestic sewage treatment area is located in Haiping Village on eastern Haimen Island (Figure 1), with an area of approximately 1600 m^2^. The domestic sewage of more than 80 households is collected into the sewage treatment system, which was built in April 2012.

### 2.2. Sewage Treatment Process

According to the test results of sewage from the septic tanks of some residents in the collection area, the concentrations of chemical oxygen demand (COD_Cr_), ammonia nitrogen (NH_3_-N), total nitrogen (TN), and total phosphorus (TP) were relatively high. Therefore, a combined treatment process involving a pretreatment, a constructed wetland, and an ecological pond was adopted. The designed sewage treatment capacity was 34 t/day.

**Figure 2:**
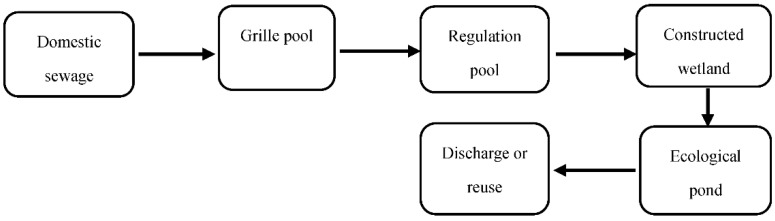
Sewage treatment process: Domestic sewage, grille pool, regulation pool, constructed wetland, ecological pond, and discharge or reuse.

The sewage treatment process is shown in Figure 2. First, the sewage collection pipe network was installed. The domestic sewage is collected into the grille pool through the automatic flow of the pipe network. Large solid matter in the sewage is intercepted by the coarse grille, and the effluent enters the regulation pool, which is composed of sedimentation pool I, the anaerobic pool, and sedimentation pool II. Packing is suspended in the anaerobic tank as a carrier for the attachment of microorganisms to improve the treatment efficiency. The hydraulic retention time is at least 10 h. The above processes are collectively referred to as the pretreatment.

After the pretreatment, the sewage enters the vertical subsurface flow constructed wetland. The constructed wetland, which utilizes the synergistic effect of substrates, plants, and microorganisms to perform secondary purification of sewage and create an ecological landscape, is the main part of the sewage treatment project. The area of the constructed wetland is 160 m^2^, the effective depth is 1.2 m, and the residence time is more than 2 d. The impermeable membrane, gravel, shell, clay, zeolite, and sand layer are laid in the constructed wetland from bottom to top. The plant species planted in the constructed wetland include *Canna indica*, *Arundo donax*, *Iris tectorum*, *Cyperus involucratus*, and *Acorus calamus*, with a planting density of 12–16 plants/m^2^.

Finally, the sewage enters the ecological pond, where it is subjected to advanced treatment. The ecological pond replaced the original sewage pond, which was reconstructed to remove silt in this experiment. The pond has a water surface area of 1100 m^2^, an effective depth of 1.5 m, and a hydraulic retention time of approximately 67 d. An ecological floating bed of 100 m^2^ was installed in the pond, and the plant species and density planted in the floating bed are the same as those in the constructed wetland.

### 2.3. Measurement Parameters and Time

The measurement parameters included dissolved oxygen (DO), DO saturation, COD_Cr_, biochemical oxygen demand (BOD_5_), suspended solids (SS), NH_3_-N, TN, TP, fecal coliform, bacteria, and viruses.

During the commissioning stage, the water quality of the influent, regulation pool effluent, constructed wetland effluent, and ecological pond effluent was measured once a month.

On the day of project acceptance, water samples were collected every 2 h, 4 times a day, beginning in the morning. The sampling locations included the water inlet and the outlet of the regulation pool, constructed wetland, and ecological pond.

### 2.4. Measurement Methods

1. Measurement method of COD_Cr_: Excessive potassium dichromate solution was added to the water samples under strongly acidic solution conditions, and then heated for 2 h. The excessive potassium dichromate was dropped with ammonium ferrous sulfate solution.
2. Measurement method of BOD_5_: The samples were cultured in a constant temperature incubator (20 ± 1 °C) for 5 days, and the dissolved oxygen concentration before and after culture was measured. The difference between the two was BOD_5_ value.
3. The TN was measured by spectrophotometry after alkaline potassium persulfate digestion.
4. The TP was measured by molybdenum antimony spectrophotometry after potassium persulfate digestion.
5. NH_3_-N was analyzed by spectrophotometry using Nessler’s reagent;
6. Water samples were filtered through a 0.45 μm filter membrane, and the filter membrane was placed on the surface of the medium. Fecal coliform was counted after 24 h of culture in the incubator at 37 °C.
7. Bacteria and viruses were filtered through a 10 μm silk filter and analyzed by flow cytometry.

## 3. Results

### 3.1. Changes in Water Quality during the Commissioning Stage

During almost three months of commissioning, the plants in the constructed wetland and ecological pond gradually grew in June and began to have a certain treatment effect. Subsequently, with the gradual formation and stabilization of the active microbiota in the sewage treatment system and resulting biological membrane, the treatment efficiency gradually increased, and the water no longer had an odor or bred mosquitoes. The monitoring results from June to August are shown in Table 1. By August 28, the concentrations of COD_Cr_ and NH_3_-N in the effluent of the ecological pond were close to those of the level I B emission standards for urban sewage treatment plants (GB18918-2002, China).

However, between August and September, the transparency of the ecological pond decreased to approximately 40 cm, and the color of the water body turned green. The DO concentration of the water body was greater than 15 mg/L, the DO saturation was greater than 190% during the daytime, and the water body of the ecological pond was in a state of algal bloom. The dominant algae were *Kirchneriella contorta,* with a density of 2.5 × 10^7^ cells/L. To control the excessive reproduction of microalgae in the ecological pond, an oxygenator was installed in the middle water surface of the ecological pond to promote the flow of water in the pond on 1 September; 600 silver carp, 300 bighead carp, and 100 black carp were released into the pond to feed on and control the amount of microalgae; floating plants, such as *Nymphaea tetragona* were planted on the water surface to reduce direct sunlight; and the reproduction of microalgae was further limited by reducing the photosynthetic intensity. By the end of September, the density of microalgae in the ecological pond had gradually decreased, the water body gradually cleared, and various indicators gradually improved.

### 3.2. Monitoring Results of Project Acceptance

**Figure 3:**
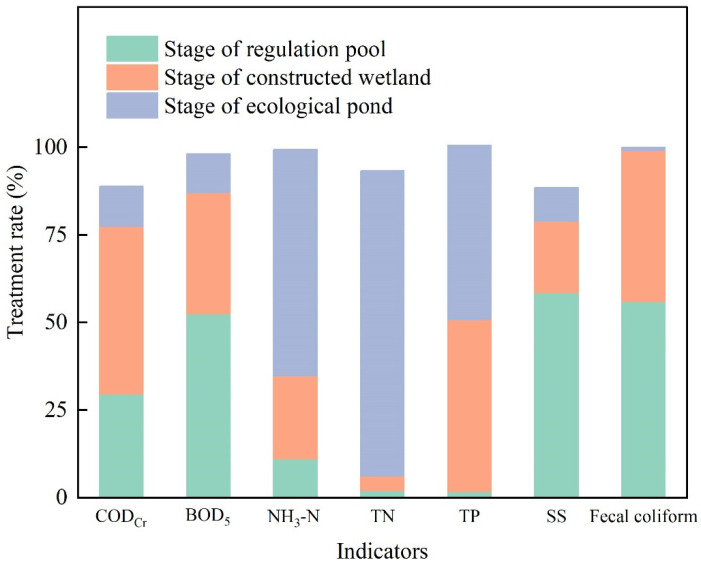
Treatment rate of pollutants at each stage of the sewage treatment project.

After October, the density of microalgae in the ecological pond further decreased, the transparency of the water increased to a depth of 1 m, and the overall water quality continued to improve. On 11 October, acceptance monitoring was performed. The average concentrations and treatment rates of the main indicators in the influent and effluent waters are shown in Table 2 and Figure 3, respectively. The COD_Cr_, BOD_5_, SS, NH_3_-N, TN and TP of the ecological pond effluent met the level I B emission standards for urban sewage treatment plants (GB18918-2002, China). The treatment rates of BOD_5_, NH_3_-N, TN and TP surpassed 95%, and the treatment rates of COD_Cr_ and SS surpassed 88%. The fecal coliform had not yet met the discharge standard of 10^4^ coliforms/L, but it was close to the discharge standard, and the removal rate had surpassed 99.97%, demonstrating a favorable treatment effect without the use of disinfectant sterilization.

**Figure 4:**
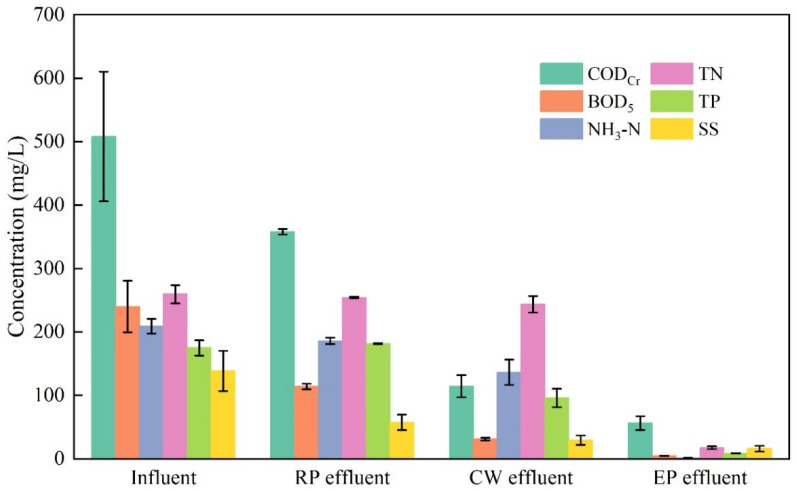
Changes in the concentrations of COD_Cr_, BOD_5_, NH_3_-N, and SS at different treatment stages (RP: regulation pool; CW: constructed wetland; EP: ecological pond).

**Figure 5:**
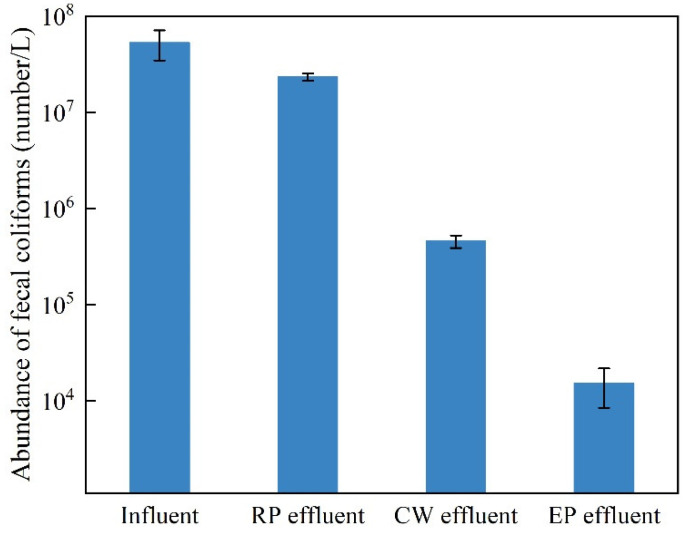
Changes in the abundance of fecal coliform at different treatment stages (RP: regulation pool; CW: constructed wetland; EP: ecological pond).

The changes in the concentrations of pollutants in the influent and effluent at each stage of sewage treatment are shown in Figure 4 and Figure 5. In general, the indicators of the various pollutants gradually decreased, but the treatment effects on the different pollutants differed in the different treatment stages.

After the two sedimentations in the regulation pool, the treatment effect on SS was the highest, with a treatment rate of 58.48%. In addition, the regulation pool also had favorable treatment effects on fecal coliform and BOD_5_, reaching 56.03% and 52.50%, respectively. However, the treatment effect of the regulation pool on N and P was relatively poor.

In the constructed wetland stage, the treatment effects on the TP, COD_Cr_ and fecal coliform were good, with treatment rates of 49.04%, 47.93%, and 43.11%, respectively, and some treatment effects on BOD_5_, NH_3_-N and SS were observed. However, the treatment effect on the TN was poor (only 4.14%).

In the ecological pond stage, the treatment effects on N and P were the highest. The treatment rates of the TN, NH_3_-N, and TP reached 87.11%, 64.61%, and 49.79%, respectively; some treatment effects were observed for other pollutants.

**Figure 6:**
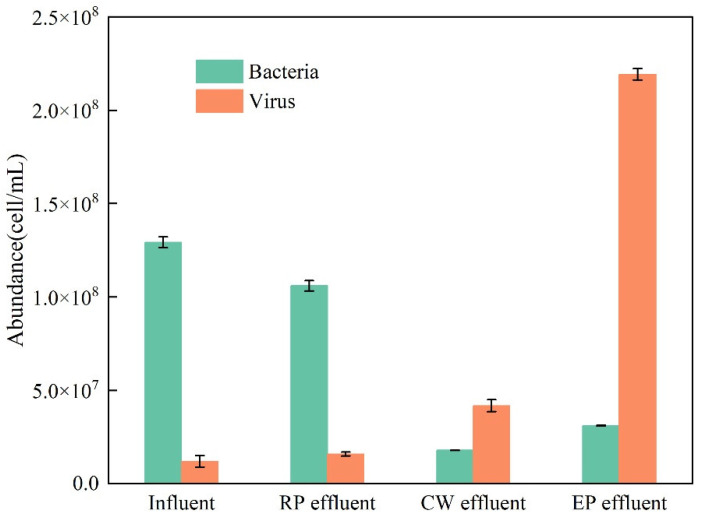
Changes in the abundance of bacteria and viruses at different treatment stages (RP: regulation pool; CW: constructed wetland; EP: ecological pond).

The changes in the abundance of bacteria and viruses in the influent and effluent of the various sewage treatment stages are shown in Figure 6. The bacterial abundance was consistent with that of fecal coliform in both the influent and effluent of the constructed wetland. In particular, the bacterial abundance decreased significantly after the constructed wetland treatment. However, the bacterial abundance in the ecological pond effluent increased. The viral abundance, in contrast, generally increased.

**Figure 7:**
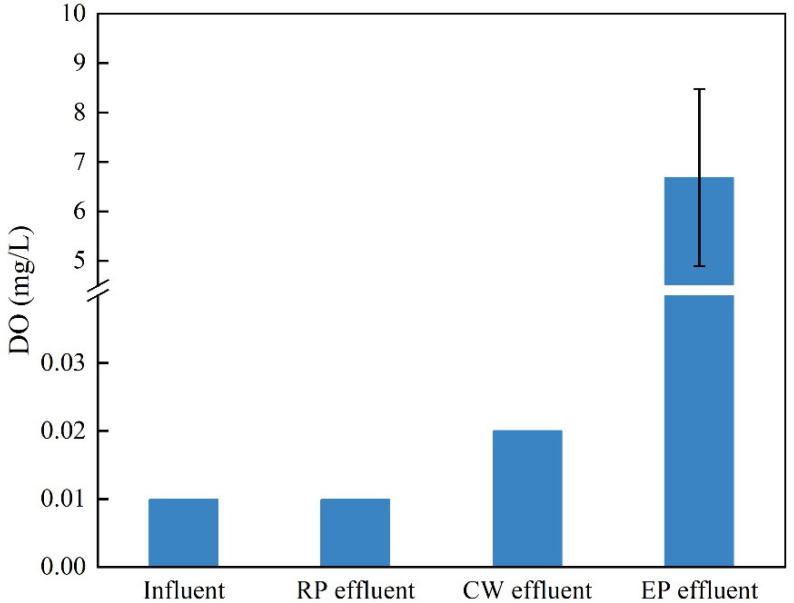
Changes in the DO concentration at different treatment stages (RP: regulation pool; CW: constructed wetland; EP: ecological pond).

The changes in the DO concentration in the influent and effluent at each stage are shown in Figure 7. In the influent and effluent of the constructed wetland, the DO concentration in the sewage was extremely low, ranging from 0.01 to 0.02 mg/L, indicating an anaerobic state. After the sewage entered the ecological pond, the DO concentration increased significantly to 6.68 mg/L.

### 3.3. Improvement of Living Environment

**Figure 8:**
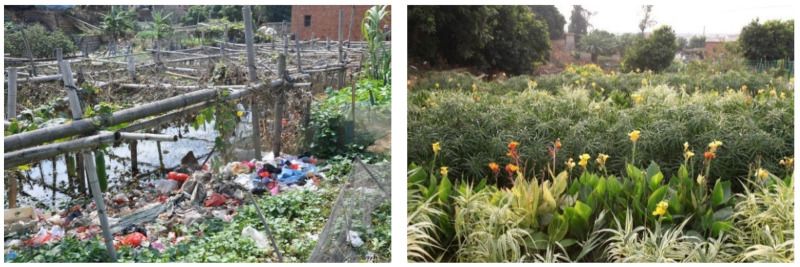
Pond before construction (**left**) and constructed wetland after construction (**right**).

**Figure 9:**
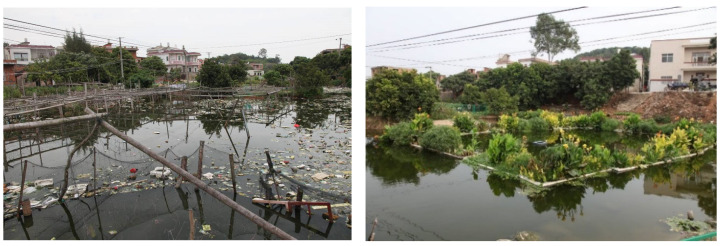
Sewage pond before construction (**left**) and ecological pond after construction (**right**).

Photos from before and after the construction of sewage treatment facility are shown in Figure 8 and Figure 9. After the sewage treatment project was completed, the domestic sewage was collected into the sewage treatment facility through the underground sewage collection pipe network, and the sanitary environment and air quality of the residential area were significantly improved. After the sewage was effectively treated, the air environment of the pond and the surrounding area was no longer malodorous and no longer bred mosquitoes, improving the living space for nearby villagers. After completion of the sewage treatment project, the surrounding landscape was also beautified. The vegetation in the constructed wetland and ecological floating beds includes various flowers, and birds such as egrets are attracted to the habitat. The post-construction landscape was greatly improved and has become a place of recreation and entertainment for nearby villagers.

The available freshwater resources on the island are extremely scarce, especially in the dry season. Before sewage treatment, island residents had to pump sewage from the pond for agriculture and even daily living needs, which had a negative impact on the health of residents, especially due to the biological toxins released by algae after algae blooms. The water quality after the treatment was greatly improved, alleviating the water shortage for nearby residents.

## 4. Discussion

### 4.1. Differences and Complementarity between the Treatment Processes of the Constructed Wetland and the Ecological Pond

Constructed wetlands are composed of plants, substrates, and microorganisms. They can effectively adsorb, enrich, and adjust soluble pollutants, such as nitrogen and phosphorus, in water through physical, chemical, and biological effects and effectively filter and precipitate insoluble pollutants [18]. The plant roots in constructed wetlands can reduce the flow velocity of water, thereby enhancing the sedimentation process [19]. The nutrient elements present in sewage are consumed by plants and stored in their tissues [20,21]. In this study, the effects of the TP, COD_Cr_, and BOD_5_ treatments were significant in the constructed wetland stage. P was adsorbed and precipitated by the substrate of the constructed wetland [22], and COD_Cr_ and BOD_5_ decreased as microorganisms in the constructed wetland decomposed pollutants.

Ecological ponds use the combined action of microorganisms and algae to treat sewage, including anaerobic and aerobic treatment processes. On the surface of ecological ponds, algae and plants in ecological floating beds produce a large amount of oxygen by photosynthesis, which is used by aerobic bacteria and facultative bacteria to oxidize and degrade organic matter and synthesize new cells. In the deep water area, the DO concentration in the water body is relatively low, and the pollutants undergo anaerobic reduction reactions under the action of anaerobic bacteria [16]. In addition, ecological floating beds share the functions of constructed wetlands. They use a suspended network composed of deep underwater tissues and roots and attached biofilms to purify sewage through biochemical, filtration, and embedding processes [23].

Therefore, the addition of an ecological pond treatment system downstream of a constructed wetland can make use of the microalgae and plants in the ecological pond to continue to absorb the NH_3_-N and inorganic phosphorus in the sewage, effectively reducing the concentration of pollutants in the water The DO concentration in the water can then be increased through the photosynthesis of microalgae and plants, thereby avoiding the direct discharge of anoxic sewage that is harmful to aquatic organisms. Simultaneously, ecological ponds can serve as a water storage unit to provide resources for the reuse of sewage and alleviate the shortage of freshwater resources on islands.

In this experiment, the concentration of NH_3_-N in the sewage was extremely high. The removal pathways of N include ammoniation, nitrification, and denitrification [9,24], of which ammoniation and nitrification require the participation of oxygen. Compared with surface flow-constructed wetlands, surface sewage flow is nonexistent in subsurface-constructed wetlands [25], which has the advantage of avoiding air pollution and minimizing mosquito breeding. However, the DO content is low in the water body of subsurface-constructed wetlands, which limits the purification capacity of constructed wetlands [26]. Adsorption by the substrate in constructed wetlands can also remove NH_3_-N, but this removal effect is relatively poor [9]. In the ecological pond stage, NH_3_-N reacts with DO in the water body to undergo an ammonia oxidation process, which can effectively remove NH_3_-N. Similarly, the removal of organic nitrogen in sewage also requires the participation of oxygen; therefore, the TN and NH_3_-N treatment effects are greater in the ecological pond than that in the constructed wetlands.

In this experiment, the abundance of fecal coliform gradually decreased as the pollutant concentration decreased in the different treatment stages. The treatment effects of the different treatment stages on fecal coliform were different, and the abundance of fecal coliform in the constructed wetland treatment stage decreased to the greatest extent. Theoretically, sufficient oxygen in an ecological pond should enable aerobic bacteria to proliferate in large quantities and subsequently inhibit the reproduction of fecal coliform bacteria. However, because the abundance of fecal coliform was significantly reduced after the constructed wetland treatment, the treatment effect of the ecological pond was not fully developed. Moreover, it should be noted that after the constructed wetland treatment, the sewage still needed to be treated in the ecological pond before meeting the discharge standards for river sewage.

Different treatment stages have different treatment effects on different pollutants, and the combination of several treatment processes has favourable effects on the treatment of various pollutants.

### 4.2. Effects of Each Treatment Stage on the Abundance of Bacteria and Viruses

In this study, the bacterial abundance generally decreased from the influent to the effluent of the constructed wetland, but the bacterial abundance in the effluent of the ecological pond increased because the first two treatment stages were essentially anaerobic environments, and the total number of bacteria gradually decreased as the concentration of pollutants decreased. The oxygen was sufficient in the ecological pond, and aerobic bacteria proliferated in large quantities, resulting in an increase in the total number of bacteria. In addition, the large number of bacteria in the ecological pond was also related to the high density of microalgae in the water. Algae blooms and deaths release large amounts of organic matter into the water, promoting the proliferation of bacteria [27,28].

Throughout the entire sewage treatment process, the viral abundance gradually increased and was the highest in the ecological pond effluent, which may have been related to the massive reproduction of microalgae and bacteria in the ecological pond, resulting in a large number of algal viruses and bacterial viruses, which in turn could inhibit the excessive reproduction of microalgae and bacteria [29,30]. This inhibitory effect also manifested in the process from the influent to the effluent of the constructed wetland, and a significant negative correlation was observed between the abundance of viruses and the abundance of bacteria.

### 4.3. Characteristics of Island Sewage and Its Influence on the Treatment Process

The domestic sewage in the rural areas of the island mainly comes from kitchens and bathrooms, with small rates of water consumption and obvious intermittent sewage discharge times [31]. Compared with sewage treatment plants, treating domestic sewage on islands by ecological treatment methods, which have lower construction costs and lower energy demands, is more suitable.

Compared with that of rural areas on the mainland, the domestic water consumption on islands is smaller due to the shortage of freshwater resources, resulting in higher concentrations of pollutants in domestic sewage. High concentrations of pollutants, such as nitrogen and phosphorus, may be related to the consumption of aquatic products, which contain high levels of protein and phosphorus, on the island. Therefore, if a constructed wetland is used alone to treat the domestic sewage of an island, the pollutant concentrations cannot meet the expected standards, so the wetland will need to be combined with an ecological pond for further treatment.

Based on field observations, the overall growth of plants in the constructed wetland was good, but the growth of *Acorus calamus* was slow, which may have been related to the high salinity and the high concentration of NH_3_-N in the domestic sewage of the island. Subsequently, the better-growing *Canna indica* and *Arundo donax* were replanted. Therefore, suitable plant species should be selected according to the environmental characteristics to ensure the constructed wetland and ecological floating bed have favorable treatment effects.

### 4.4. Effects of Control Measures on Algal Blooms in Ecological Ponds

Ponds are an important resource on islands; they provide habitats for aquatic organisms and are important natural landscapes. Therefore, restoring contaminated ponds is very important [32]. The discharge of sewage into ecological ponds may cause a large number of algae to reproduce and form algal blooms. To control the excessive reproduction of microalgae in ecological ponds, measures can be taken, such as installing ecological floating beds on the water surface, installing oxygenators, introducing fish that feed on algae, and planting *Nymphaea tetragona*. With the continuous growth of fish and plants, algal blooms can be gradually inhibited. Standing water is conducive to the rapid propagation of microalgae. The installation of an elevated fountain oxygenator can promote the microcirculation of water in ecological ponds, which controls the reproduction of algae. Algae require a certain amount of light to grow, and reducing the amount of light penetrating the water surface can inhibit algal blooms. As plants grow, the amount of direct sunlight decreases, and the reproduction of microalgae is limited as the photosynthetic intensity is reduced. In addition, algae-feeding fish can help control algae reproduction. Therefore, the construction of a plant–animal–microbe ecological chain in ecological ponds provides a new research direction for extending the service life of ecological treatment systems and improving the purification effect of the water environment [33,34].

## 5. Conclusions

The results showed that the combination of multiple ecological treatment processes had favorable treatment effects on various pollutants in the sewage, with the treatment rates surpassing 88%.

Different treatment stages have different treatment effects on different pollutants. The constructed wetland played an important role in sewage treatment, particularly for the TP, COD_Cr_, and BOD_5_. In contrast, the treatment effect was the highest for the TN and NH_3_-N in the ecological pond. The treatment effects on the fecal coliform in the constructed wetland and ecological pond were very high.

In future applications, it is very important to maintain the balance of constructed wetland and ecological pond ecosystems to ensure the long-term effectiveness of ecological treatments.

## Figures and Tables

**Table 1:** Water quality monitoring results during the commissioning stage of the sewage treatment project (mg/L).

Date	Influent		RP Effluent		CW Effluent		EP Effluent	
	COD_Cr_	NH_3_-N	COD_Cr_	NH_3_-N	COD_Cr_	NH_3_-N	COD_Cr_	NH_3_-N
22 June	390.2	128.1	280.4	97.5	128.3	38.5	87.6	9.1
16 July	374.4	143.5	317.6	122.3	148.6	63.2	67.3	15.7
21 August	204.2	76.6	162.7	52.9	81.3	31.1	69.8	16.2
28 August	192.1	68.7	172.5	57.3	87.3	37.4	60.2	8.6

(RP: regulation pool; CW: constructed wetland; EP: ecological pond).

**Table 2:** Average concentrations of the main indicators in the influent water and effluent water of each treatment stage.

Sample	COD_Cr_ (mg/L)	BOD_5_ (mg/L)	NH_3_-N (mg/L)	TN (mg/L)	TP (mg/L)	SS (mg/L)	Fecal coliform (Number/L)
Influent	508.0	240.0	209.0	259.50	18.48	138.5	5.29 × 10^7^
RP effluent	358.0	114	185.75	254.25	18.15	57.5	2.33 × 10^7^
CW effluent	114.5	31	136.25	243.5	9.58	29.25	4.55 × 10^5^
EP effluent	56.5	4.55	1.22	17.45	0.88	16.0	1.51 × 10^4^
Standard I B (GB18918-2002)	60	20	8	20	1	20	10^4^

(RP: regulation pool; CW: constructed wetland; EP: ecological pond).
